# Role of Indocyanine Green in Diagnosing Diabetic Ulcer: A Case Report

**DOI:** 10.7759/cureus.54905

**Published:** 2024-02-25

**Authors:** Aditi V Rokade, Abhay Mudey, Prishita Gupta, Nachiket P Rahate

**Affiliations:** 1 Community Medicine, Jawaharlal Nehru Medical College, Datta Meghe Institute of Higher Education and Research, Wardha, IND

**Keywords:** diabetic foot, ulcer, near infrared, diabetes mellitus, indocyanine green

## Abstract

Diabetic foot is a syndrome complex that presents with a serious complication arising from a diabetic ulcer. Poor blood circulation and nerve injury (neuropathy), especially in the limbs, can result from persistently elevated blood sugar levels. These elements play an important role in the formation of diabetic ulcers, which frequently occur on the feet and are prone to infection and sluggish healing. These ulcers have the potential to worsen and develop diabetic foot, a more serious ailment if left untreated. Diabetic foot issues can lead to increased vulnerability to infections, gangrene, and in severe cases, amputation. Diabetes patients may fail to notice small wounds or infections because of reduced feeling from neuropathy, which can lead to more serious problems. Moreover, decreased blood flow makes it more difficult for the body to mend itself, which makes diabetic foot issues more difficult to manage and cure. Glycemic management, timely medical intervention, and routine foot care are essential for preventing and lessening the severe effects of diabetic foot. We herein highlight the case of a 57-year-old male with a traumatic diabetic foot in whom indocyanine green (ICG) dye was used to identify the uptake of the involved tissue. The aspect of this case is the late presentation of a patient with uncontrolled diabetes. Further, we can better manage the patient in the preoperative phase by using ICG.

## Introduction

The burden of diabetes is increasing and high in developing countries like India, and it is mainly encouraged by the increasing prevalence of unhealthy diets, lack of exercise, overweight, etc. According to projections, 77 million people in India had diabetes in 2019, and by 2045, that number is predicted to reach over 134 million. About 57% of these people never receive a diagnosis [1]. Moreover, the patients with diabetes are immunocompromised and even the slightest trauma can go unnoticed due to neuropathy and subsequently lead to the development of ulcers and widespread infection. Minor foot trauma can go undetected due to a lack of sensation caused by peripheral neuropathy from things like badly fitted shoes or injuries. This trauma may cause ulceration and subsequent infection if it is repeated or ignored. Motor neuropathy resulting from myelinated fiber loss causes muscle atrophy, foot deformities, and irregularities in gait. These biomechanical alterations increase shearing and pressure, which predisposes neuropathic people to develop foot ulcers [2]. A fluorescent dye called indocyanine green (ICG) has been widely employed in bioimaging procedures recently to help identify obscure and frequently difficult-to-find anatomical features. To get around these challenges during surgery, this way, when dissecting the organ, they can prevent damaging the vital systems that are close to the site of dissection. By helping to see the tissues at depth and in the dissection area, near-infrared (NIR) imaging helps to avert any potential surgical disasters caused by the tissues unintentionally harming nearby important structures [3]. New intraoperative imaging systems that take advantage of the NIR light spectrum have been assessed clinically in recent years for various uses because NIR light can reach up to 10 mm into tissue and is more favorable for intraoperative purposes than visible light which has a wavelength between 700 and 900 nm. These days, only two of them - methylene blue (MB) and ICG - remain [4]. ICG is a tricarbocyanine dye which is a fluorescent, nontoxic dye with an absorption spectrum of 790nm [5]. NIR requires a fluorophore, such as ICG dye, and an imaging device (scope and camera system) that can identify and excite the fluorophore [6]. In this report, we present a case of a 57-year-old male with a history of diabetic ulcer on his foot due to trauma and the use of ICG in the diagnosis of the same.

## Case presentation

A 57-year-old male presented to the emergency department with a known history of diabetes mellitus (DM) with a referral from the rural hospital for an ulcer. The patient gave a history of getting a cut on his foot from a sharp stone while walking barefoot that he did not initially feel over a month ago. A few weeks after the cut, as the wound progressed, he noticed a wound on his right foot, bloody discharge, and pain in the ankle. The patient subsequently presented to a rural hospital where the wound was taken care of and sent to our institution for further management. The patient has been diabetic for 10 years and has been taking metformin, however, he has not been compliant with his medication for one and a half years. The patient has a family history of DM, his father had DM. The patient does not have any drug allergies. The patient is a nonsmoker and nonalcoholic. He can walk but complains of ankle pain. The patient has no history of palpitations, shortness of breath, or chest pain.

**Figure 1:**
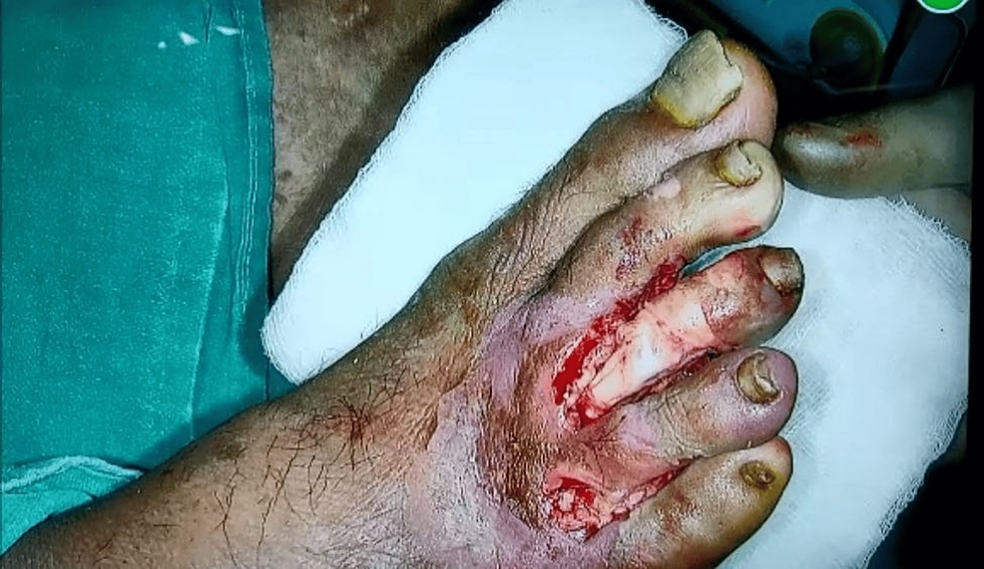
Ulcer over the distal dorsum of the right foot

The patient's vital records included a temperature of 97.4 degrees Fahrenheit, pulse of 110 beats per minute, respiratory rate of 18 breaths per minute, and blood pressure (BP) of 138/90mmHg, and random blood sugar test revealed a value of 298mg/dL. On examination of his right foot, the skin was erythematous, and tender to touch. There was an ulcer between the second and third toe and the fourth and fifth toe of the right foot (Figure 1).

**Figure 2:**
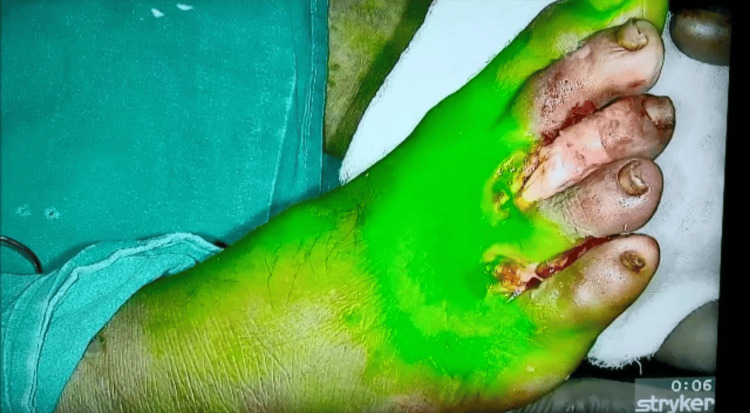
Indocyanine green dye injected

The patient had decreased pinprick sensation on the plantar surface of the right foot although vibration sense is intact. All pulses were intact. Laboratory findings revealed elevated fasting blood glucose levels of 128mg/dL, HbA1c of 7.2% random blood glucose level of 298mg/dL, and hemoglobin of 10.8g/dL. As a part of routine investigations, he had an electrocardiogram (ECG) and a chest x-ray, which were normal. After a thorough explanation of the procedure to the patient, we acquired consent for the procedure as a part of the preoperative procedure to use ICG in identifying viable tissues. Initially, a preliminary dosage of ICG is administered to assess any potential reactions in the patient. Subsequently, the patient is monitored for any adverse effects. Following confirmation of stable vital signs, a 0.1mg/kg dose of 0.1% ICG is intravenously injected 30-60 minutes before the procedure to evaluate tissue viability as shown in Figure 2.

**Figure 3:**
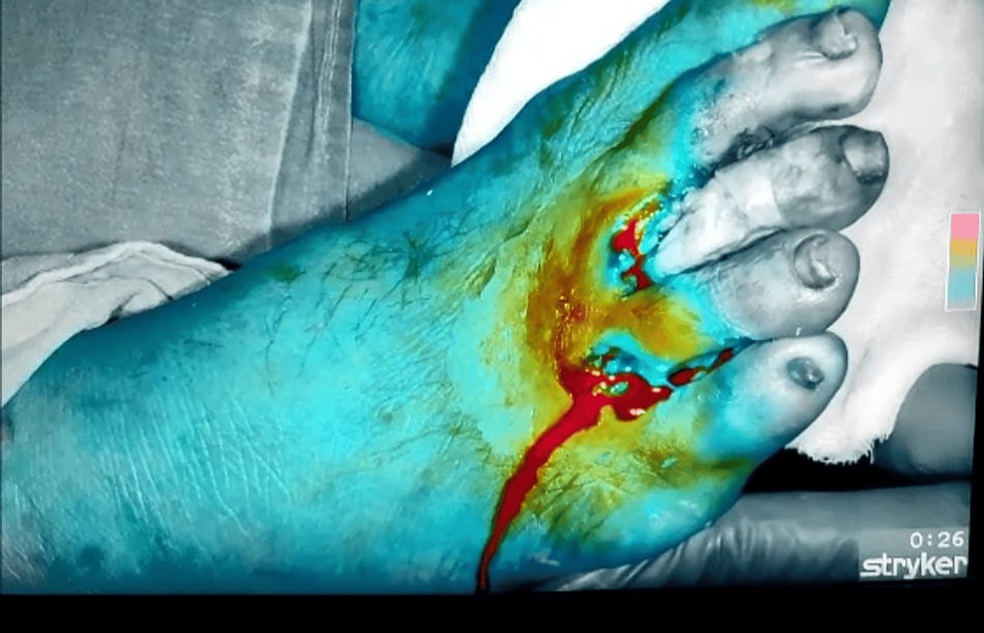
Poorly perfused second, third, and fourth toe appear in gray color.

**Figure 4:**
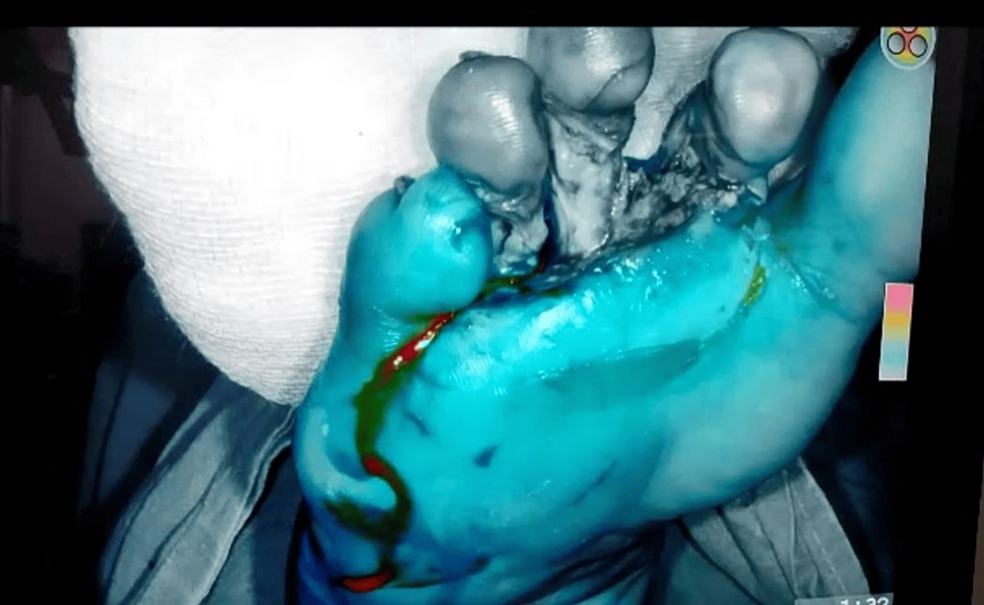
Poorly perfused second, third, and fourth toe appear gray color.

After initiating the surgery, we flipped it to NIR imaging mode because of which tissues at the depth of 10mm were visible. Within 15 seconds of administration of dye, the blood vessels can be visualized and NIR can differentiate a tissue with poorly, moderate, or healthy blood supply. The poorly perfused area has to be operated again so that it can be amputated accordingly. Figures 3, 4 show the gray area which is poorly perfused with blood supply and will be eventually amputated.

## Discussion

Diabetic foot ulcers (DFU) are a significant global health issue. An occurrence that could be avoided, like a mild trauma, typically has far-reaching consequences. DM continues to be the leading cause of nontraumatic lower limb loss globally. Men have a greater prevalence of DFUs than women. The most common complications are peripheral arterial disease and diabetic neuropathy [7]. Large prospective studies have demonstrated that individuals with type 1 and type 2 diabetes can postpone the start and progression of diabetes when they receive intensive glycemic control aimed at achieving near-normoglycemia [8]. In addition to medication, proper foot evaluation at least annually, having a multidisciplinary approach like proper diet, cessation of smoking, wearing therapeutic footwear which is plantar pressure-based, and wound care should be done. Vascular assessment of the peripheral pulses should also be employed [8].

The use of ICG is historical and has been on the medical stage for more than 50 years. Emission spectra in the NIR (approximately 700-850 nm) are used along with ICD since they can excite laser diodes at a low cost, reduce dispersion, boost tissue penetration depth, and absorb and illuminate biological samples with minimal interference and hence fluorophores have attracted a lot of interest in a variety of biomedical fields [9]. The main characteristic of optical fluorescence of ICG is its main use in medical settings. It was first used for liver function evaluation. ICG does not pass through the digestive system and when injected intravenously is quickly conjugated by plasma protein, primarily serum albumin, and then excreted in its unconjugated form by the liver through secretion into the biliary system. ICG has demonstrated exceptional optical qualities that have not been surpassed by emerging technologies because it was introduced in the clinical field like in diagnosing tissues with moderate, mild, or poor tissue perfusion. ICG's extensive use, short half-life of less than five minutes, and high safety dose have made it more applicable in a variety of novel clinical domains, including photodynamic treatment, photoacoustic imaging, and reporter systems [10]. ICG is not frequently linked to negative responses. ICG has a long history of usage, and a good safety index, and is rarely associated with allergies. It is also known to have uncommon incidences of anaphylaxis, syncope, urticaria, and vasovagal response. It is known that when ICG is exposed to UV light, it decomposes into toxic waste products that result in a wide variety of unknown chemicals. Some reported mild side effects, such as hot flashes and sore throats [11]. The identification of subcutaneous perforations and microvessels in tissues up to 20 mm was greatly facilitated by ICG fluorescence imaging. The surgeons were able to select healthy, viable tissues with sufficient anastomosis because of this. In the microvessels, it also aided in the detection of any recently developed iatrogenic thromboses [3].

## Conclusions

In this case, a 57-year-old male who has had a history of diabetes for 10 years and is non-compliant with medication, presented with chief complaints of a wound, and pain over the right foot after walking barefoot. Complete blood count and random blood sugar pointed towards the diagnosis of diabetic foot. This case describes the usual presentation and diagnostic findings of DFU. The etiology is multifactorial with the most important here being noncompliance with oral hypoglycemic agents. For further management, ICG was used for identifying and differentiating tissues with poor perfusion from the normal tissues and eventually amputating the affected sites to prevent the complication of necrosis. Further investigations are required to minimize any form of deformity in the patient.
